# Oxygen overload

**DOI:** 10.7554/eLife.75695

**Published:** 2021-12-22

**Authors:** Britta Foerster

**Affiliations:** 1 Research School of Biology, Australian National University Canberra Australia

**Keywords:** Pyrenoid, hyperoxia, hydrogen peroxide, photosynthesis, carbon dioxide concentrating mechanism, *Chlamydomonas reinhardtii*

## Abstract

A structure that helps algae photosynthesize when carbon dioxide levels are low may also play a role during hyperoxia conditions.

**Related research article** Neofotis P, Temple J, Tessmer OL, Bibik J, Norris N, Poliner E, Lucker B, Wijetilleke S, Withrow A, Sears B, Mogos G, Frame M, Hall D, Weissman J, Kramer DM. 2021. The induction of pyrenoid synthesis by hyperoxia and its implications for the natural diversity of photosynthetic responses in *Chlamydomonas*. *eLife* **10**:e67565. doi: 10.7554/eLife.67565

Bioreactors of algae are commonly used to generate biomass, a renewable source of energy. To do this, scientists exploit the algae’s ability to efficiently convert carbon dioxide into organic compounds, such as sugar, using photosynthesis. This process is known as carbon fixation and requires high levels of inorganic carbon in order to be successful. If the primary enzyme involved in this reaction – called Rubisco (short for ribulose-1,5-bisphosphate carboxylase/oxygenase) – does not receive enough carbon dioxide, it mistakenly fixes oxygen instead. This results in unfavourable by-products that inhibit photosynthesis and require lots of energy to recycle (Peterhansel et al., 2010).

To overcome this problem, algae evolved a carbon dioxide concentrating mechanism (CCM) that allows the rate of photosynthesis to remain high despite low levels of inorganic carbon (Badger and Price, 1992; Raven et al., 2017). Much of what is known about the CCM has come from studying a single cell alga known as *Chlamydomonas* (Mackinder et al., 2017). These studies revealed two key components that allow the CCM to increase the concentration of carbon dioxide around Rubisco enzymes: transporter proteins that pump carbon dioxide into the chloroplast, and structures called pyrenoids which sequester Rubisco enzymes. Pyrenoids are condensed proteins which are often surrounded by a starch sheath that promotes the selective entry of carbon dioxide and shields Rubisco from oxygen. However, the mechanisms and signals underlying the formation of these pyrenoids have largely remained elusive.

Past and current research has mostly focused on pyrenoids that appear when CCMs are induced by low levels of inorganic carbon (Wang et al., 2015). Now, in eLife, David M Kramer (Michigan State University) and colleagues – including Peter Neofotis (also from Michigan State) as first author – report that pyrenoids may also be induced by high levels of oxygen, in the absence of other CCM components (Neofotis et al., 2021).

The team – which included researchers from Michigan State and ExxonMobil – found that a species of alga called *Chlamydomonas reinhardtii* contains pyrenoids when there are extremely high levels of oxygen in the air (also known as hyperoxia) despite carbon dioxide levels also being high (Figure 1). The structure of these oxygen-induced pyrenoids is strikingly similar to those formed in response to low levels of inorganic carbon. This led Neofotis et al. to hypothesize that the signal that induces pyrenoid formation may be produced when photosynthesis takes place either under hyperoxia conditions or when there are low levels of carbon dioxide.

**Figure 1.**
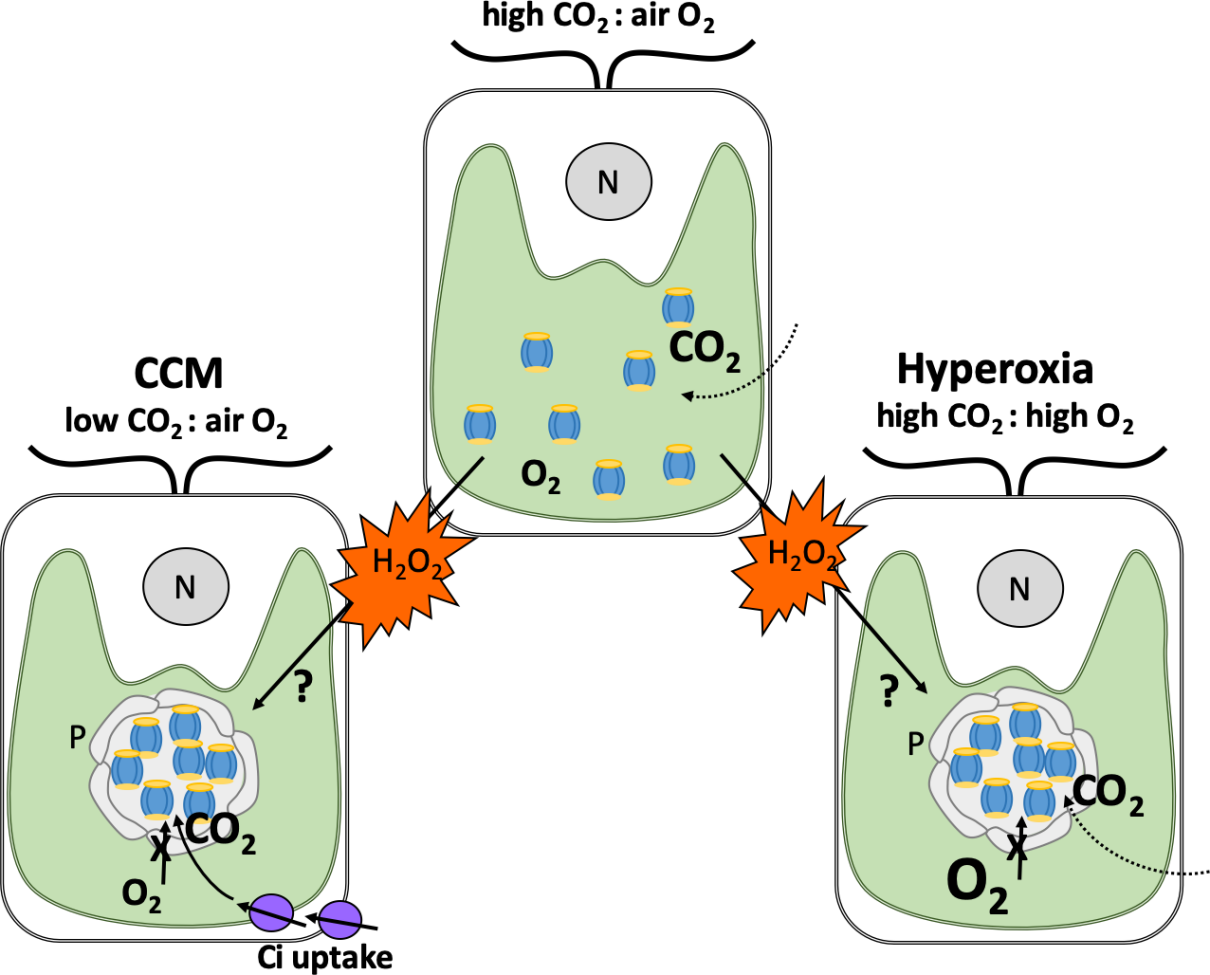
How hydrogen peroxide stimulates the formation of pyrenoids under different conditions. Depicted are typical *Chlamydomonas* cells with a single cup-shaped chloroplast (green) and a nucleus (grey circle, N). When carbon dioxide (CO_2_) levels in the air surrounding the alga are higher than oxygen (O_2_; top), Rubisco enzymes (blue and yellow) are dispersed in the chloroplast and efficiently fix carbon dioxide. When carbon dioxide levels are lower than oxygen (left), Rubisco fixes oxygen instead. This leads to harmful metabolites that need to be recycled, resulting in the generation of the by-product hydrogen peroxide (H_2_O_2_; orange). To counteract this, *Chlamydomonas* employ a carbon dioxide concentrating mechanism (CCM) which sequesters Rubisco enzymes in to pyrenoids that preclude the entry of oxygen (grey circular structure), and produces proteins that transport carbon dioxide into the chloroplast (Ci; purple). When oxygen levels are high (also known as hyperoxia; right), this leads to oxygen fixation and production of hydrogen peroxide as well. Neofotis et al. found that *Chlamydomonas* also contain pyrenoids under these conditions, even when there are high amounts of carbon dioxide and the full CCM is suppressed. This led them to propose that hydrogen peroxide triggers the formation of pyrenoids via an unknown mechanism when oxygen levels are high and when there are insufficient amounts of carbon dioxide.

Image credit: Britta Foerster (CC BY 4.0)

The signalling molecule hydrogen peroxide was considered to be the strongest candidate for the role as it is a by-product of oxygen fixation, a scenario that can occur when oxygen levels are high or when there are insufficient amounts of carbon dioxide (Figure 1). Further experiments showed that hydrogen peroxide levels and the formation of pyrenoids are highly correlated, providing strong evidence for this hypothesis.

To address the role pyrenoids play under high oxygen stress, Neofotis et al. studied and compared *Chlamydomonas* strains which have varying tolerance to hyperoxia. The experiments showed that the pyrenoids found in these genetic variants have different structures and sometimes fail to form a sheath. Algae that could endure high levels of oxygen and formed fully starch-sheathed pyrenoids had significant growth advantages over those that had more fragmented and poorly developed sheaths. This is consistent with the theory that pyrenoids also help to enhance photosynthesis under hyperoxia conditions, in addition to their role in the CCM.

Many questions remain to be answered to fully understand how hydrogen peroxide signalling leads to the formation of pyrenoids, regardless of whether low levels of inorganic carbon or hyperoxia are the eliciting cue. It is possible that other signals may affect the formation of pyrenoids, perhaps in response to other environmental stresses. Furthermore, how pyrenoids assemble and the specific roles of each of their different parts still remain to be explored. Nevertheless, the work of Neofotis et al. sets the scene for new, exciting avenues of research that may lead to the development of more efficient algae that can be used in bioreactors and other biotechnological applications.
